# Neural Correlates of Working Memory Maintenance in Advanced Aging: Evidence From fMRI

**DOI:** 10.3389/fnagi.2018.00358

**Published:** 2018-11-06

**Authors:** Maki Suzuki, Toshikazu Kawagoe, Shu Nishiguchi, Nobuhito Abe, Yuki Otsuka, Ryusuke Nakai, Kohei Asano, Minoru Yamada, Sakiko Yoshikawa, Kaoru Sekiyama

**Affiliations:** ^1^Division of Cognitive Psychology, Faculty of Letters, Kumamoto University, Kumamoto, Japan; ^2^Department of Behavioral Neurology and Neuropsychiatry, United Graduate School of Child Development, Osaka University, Suita, Japan; ^3^Division of Human and Social Sciences, Graduate School of Social and Cultural Sciences, Kumamoto University, Kumamoto, Japan; ^4^Department of Human Health Sciences, Graduate School of Medicine, Kyoto University, Kyoto, Japan; ^5^Kokoro Research Center, Kyoto University, Kyoto, Japan; ^6^Graduate School of Advanced Integrated Studies in Human Survivability, Kyoto University, Kyoto, Japan

**Keywords:** aging, fMRI, over-recruitment, compensation, working memory, maintenance, prefrontal

## Abstract

Working memory (WM)-related brain activity is known to be modulated by aging; particularly, older adults demonstrate greater activity than young adults. However, it is still unclear whether the activity increase in older adults is also observed in advanced aging. The present functional magnetic resonance imaging (fMRI) study was designed to clarify the neural correlates of WM in advanced aging. Further, we set out to investigate in the case that adults of advanced age do show age-related increase in WM-related activity, what the functional significance of this over-recruitment might be. Two groups of older adults – “young–old” (61–70 years, *n* = 17) and “old–old” (77–82 years, *n* = 16) – were scanned while performing a visual WM task (the *n*-back task: 0-back and 1-back). WM effects (1-back > 0-back) common to both age groups were identified in several regions, including the bilateral dorsolateral prefrontal cortex (DLPFC), the inferior parietal cortex, and the insula. Greater WM effects in the old–old than in the young–old group were identified in the right caudal DLPFC. These results were replicated when we performed a separate analysis between two age groups with the same level of WM performance (the young–old vs. a “high-performing” subset of the old–old group). There were no regions where WM effects were greater in the young–old group than in the old–old group. Importantly, the magnitude of the over-recruitment WM effects positively correlated with WM performance in the old–old group, but not in the young–old group. The present findings suggest that cortical over-recruitment occurs in advanced old age, and that increased activity may serve a compensatory function in mediating WM performance.

## Introduction

As they get older, many older adults become aware that their memory is not as good as it used to be. There is considerable evidence, however, that not all types of memory are equally vulnerable to aging. Episodic memory and working memory (WM) show relative decline with increasing age, while other types of memory (such as semantic memory, procedural memory, and priming) are relatively spared (Hultsch et al., 1992; Park et al., 2002; Nyberg et al., 2012). The age-related decline of episodic and WM seems to be gradual, but the decline accelerates after 70 years of age (e.g., Park et al., 2002). Although many functional neuroimaging studies have investigated the age-related change in brain activity that underlie decline in memory performance, the majority of these studies compared participants with a mean age of 25 with those in their mid-sixties (e.g., Daselaar et al., 2003; Sun et al., 2005; Grady et al., 2008; Nyberg et al., 2009; Duarte et al., 2010; Heinzel et al., 2017; see also Spreng et al., 2010). Hence there is less evidence regarding the neural correlates of episodic and WM in individuals aged around 80 years and older.

There are several reasons for focusing on participants aged around 80 years and older. First, given the fact that the proportion and number of individuals in this age class have been growing rapidly in the global population (World Population Prospects: The 2017 Revision^1^), clarifying the neural correlates of age-related memory decline in these elderly people is of significant importance. Second, as mentioned earlier, the decline of memory functions accelerates with increasing age (e.g., Park et al., 2002). For example, relative to individuals in their mid-sixties, 80-year-old individuals typically decline markedly in their cognitive abilities, the difference being similar to that between people in their mid-twenties and those in their mid-sixties. Contrasting brain activity of two groups of older adults (mid-sixties vs. 80-year-old adults) allows us to identify whether the results follow a similar pattern to that shown in prior studies comparing brain activity between young and older adults (see below for details). Finally, individual differences in cognitive decline become more pronounced with advanced age (de Frias et al., 2007; Habib et al., 2007; see also Morrison and Baxter, 2012; Nyberg et al., 2012). Findings from studies employing participants at a very old age reveal the neural mechanisms underlying the phenomenon that some older adults exhibit relatively stable cognitive performance that is comparable to that of younger adults, while others show a marked and more apparent decline.

With regard to episodic memory, Wang et al. (2009) compared retrieval-related activity between two groups of older adults (“young–old,” 63–77 years, and “old–old,” 84–96 years) using a yes/no recognition memory task to identify effects of advanced aging. They reported old > new effects (i.e., activity enhanced for correctly judged old items compared to correctly judged new items) common to both groups in the parietal and prefrontal cortex. No clusters were identified with greater effects in the old–old group, while the medial parietal region showed greater effects in the young–old group. Within a similar framework, in the present functional magnetic resonance imaging (fMRI) study, we focused on WM and contrasted its neural correlates in young–old and old–old adults using a visual WM task. On the basis of prior findings that WM performance declines markedly after 70 years of age (e.g., Park et al., 2002), we analyzed the data from two separate groups: individuals aged between 61 and 70 years for the young–old group, and those aged 77 years and older for the old–old group. We set a 7-year age gap between the groups, firstly because 77 years was the upper age limit of the young–old group in Wang et al. (2009), and secondly because the prevalence of mild cognitive impairment (MCI) or dementia increases non-linearly from some point between 75 and 79 years in Japan (Asada, 2013), suggesting that people at this age become more heterogeneous as a group and more susceptible to age-related decline. The young–old group was similar, in terms of its age population, to older adults employed in prior fMRI studies of aging (cf. Spreng et al., 2010).

Working memory represents a cognitive system that allows us to temporarily maintain and manipulate information (Baddeley, 1986). This function is required for a wide range of cognitive abilities, such as planning, problem solving, or reasoning. There is emerging consensus that WM maintenance is implemented by allocating attention to internal representations of an item (Cowan, 1995; Oberauer, 2002; Fuster, 2009; D’Esposito and Postle, 2015; Eriksson et al., 2015). More specifically, it is assumed that when task-relevant representations are in the focus of attention, an active maintenance process ensures maintenance through top-down signals from fronto-parietal networks to posterior regions specifically related to the current content of WM (Fuster, 2009; D’Esposito and Postle, 2015; Eriksson et al., 2015). Directly supporting this notion, neurophysiological studies in monkeys (Funahashi et al., 1989; Miller et al., 1996; see also Curtis and D’Esposito, 2003) and, more recently, functional neuroimaging studies in young human adults (Courtney et al., 1997; Leung et al., 2002; see Curtis and D’Esposito, 2003; Owen et al., 2005; Rottschy et al., 2012 for reviews) have demonstrated activity in fronto-parietal networks including the dorsolateral prefrontal cortex (DLPFC) and the lateral parietal cortex during WM maintenance (the “core” WM network; Rottschy et al., 2012).

As documented in a meta-analysis by Spreng et al. (2010; see also Rajah and D’Esposito, 2005; Reuter-Lorenz and Sylvester, 2005), relative to younger adults, older adults show either increased or decreased WM-related activity in the DLPFC and the lateral parietal cortex. Several studies have reported age-related *over*-recruitment of the DLPFC during low WM load tasks (e.g., maintenance tasks). In contrast, age-related *under*-recruitment of the DLFPC has been reported during high WM load tasks (e.g., manipulation tasks). Decreased activity in older adults compared to younger adults is usually characterized as neurocognitive decline (Grady, 2008; Grady, 2012). However, the functional meaning of age-related *increases* in neural activity (“over-recruitment”) is unclear.

Two patterns of age-related over-recruitment have been interpreted as reflecting compensatory mechanisms that support cognitive performance despite age-related decline in neural functions. First, older adults tend to recruit PFC regions contralateral to those most active in younger adults, leading to a more bilateral activation pattern (e.g., Reuter-Lorenz et al., 2000; Piefke et al., 2012; hemispheric asymmetry reduction in older adults, HAROLD; Cabeza, 2002). Second, age-related increase in PFC activity is coupled with decreased activity in posterior brain regions involved in perceptual processing (e.g., Dennis et al., 2008; Pfeifer et al., 2016; posterior–anterior shift in aging, PASA; Davis et al., 2008). However, alternative view proposed that age-related PFC over-recruitment reflects a reduction in neural specificity or efficiency with aging, and that, hence, over-recruitment reflects age-related changes in neural functions that have a negative impact on cognitive performance (e.g., Logan et al., 2002; Li et al., 2006; Morcom and Henson, 2018).

The compensatory view is often applied when, for instance, enhanced WM-related activity (such as in the DLPFC and the lateral parietal cortex) is observed in older compared to younger adults, even when performance levels on WM tasks are matched (e.g., Cabeza et al., 2004; Mattay et al., 2006). Alternatively, it is applied when WM-related activity in over-recruited regions positively correlates with better WM performance in older but not in younger adults (e.g., Reuter-Lorenz et al., 2000; Sun et al., 2005; Nyberg et al., 2014). Both cases indicate that over-recruitment reflects the engagement of processes beneficial to performance. Thus, to interpret the functional significance of age-related over-recruitment, it is crucial to examine the relationship between brain activity and task performance (Grady, 2008; Grady, 2012).

Only few studies have investigated neural correlates of WM including older groups of participants consisting of individuals around the age of 80. In one study, Nyberg et al. (2014) investigated age-related changes in brain activation among four age groups (25–50, 55–60, 65–70, and 75–80 years) using verbal WM tasks. In this study, participants maintained four target letters in memory and then compared them to a probe letter during a maintenance task, or generated letters following two target letters in the alphabet, and kept those in memory, during a manipulation task. The authors found age-related linear decreases in activity in the bilateral DLPFC and the lateral parietal cortex during the manipulation task (high WM load), and a linear increase in the left DLPFC during the maintenance task (low WM load). In addition, within the oldest group (75–80 years), higher left DLPFC recruitment was associated with better performance. There was no such tendency in the youngest group (25–50 years). However, this study did not directly compare the oldest group (75–80 years) with the other older groups of participants. In addition, since this study only used verbal material as stimuli, the neural correlates of visual WM in individuals around the age of 80 are still unknown. Given the fact that visual WM abilities are more vulnerable to aging than verbal WM (Reuter-Lorenz and Sylvester, 2005), the use of visual material may reveal the effect of advanced aging more clearly.

The purpose of the present fMRI study was twofold. First, we investigated whether old–old adults demonstrate increased WM-related activity relative to young–old adults, that is, a similar pattern of “age-related over-recruitment” when comparing activity of older to that of younger adults. Second, we set out to investigate, in the case that old–old adults do show age-related increases in neural activity, what the functional significance of this over-recruitment might be. Thus, two groups of older adults (young–old and old–old) were scanned while performing a visual WM task (the *n*-back task: 0-back and 1-back). We contrasted the neural correlates of WM between the two age groups, and then further investigated the relationship between WM-related activity and task performance.

The *n*-back task is the most commonly used experimental paradigm for functional neuroimaging of WM (e.g., Grady et al., 2008; Nyberg et al., 2009; Stanley et al., 2015; Heinzel et al., 2017; see Owen et al., 2005; Rottschy et al., 2012 for reviews); during this task, participants are asked to monitor a series of stimuli and to judge whether the currently presented stimulus is the same as the one presented *n* trials before. This paradigm allows manipulation of WM load by increasing or decreasing *n*. We only used a 1-back task, which is widely considered a maintenance task with low WM load, for two reasons. Firstly, because we wanted to focus on age-related *increases* in WM-related activity (see above), and, secondly, because data from a previous behavioral study by our group (Kawagoe and Sekiyama, 2014) revealed that 2-back tasks for face and location may be too cognitively demanding for old–old adults [the mean percentage of correct responses was 50% (chance level), collapsing across face and location conditions].

## Materials and Methods

### Participants

The present results are originally based on the data from 18 young–old adults (11 female; age range = 61–70 years; mean age = 66.2 years) and 24 old–old adults (10 female; age range = 77–91 years; mean age = 79.8 years), recruited via the Kyoto-city Silver Human Resources Center, Japan. All participants were right-handed, had normal or corrected-to normal vision, no history of neurological, cardio-vascular, or psychiatric illness, and no contraindications for MR imaging. Informed consent was obtained from all participants in accordance with the requirements of the Ethical Committee of Kumamoto University, which approved the study. Nine participants were excluded from the analyses. Four old–old adults were excluded because of abnormalities in their anatomical scan, and the remaining five (four old–old adults and one young–old adult) were excluded because they scored below 24 on the Mini-Mental State Examination (MMSE), and/or more than 1.5 standard deviations (SD) below their age-appropriate mean on the Wechsler Memory Scale-Revised (WMS-R) Logical Memory II. Because the Japanese version of the WMS-R manual does not provide standardized means for participants above the age of 75, age means were obtained from Kawano (2012). Thus, data from 17 young–old (10 female; age range = 61–70 years; mean age = 66.1 years) and 16 old–old (seven female; age range = 77–82 years; mean age = 79.4 years) are reported here.

### Neuropsychological Testing

A battery of standardized neuropsychological tests was administered to all participants in a session separate from the fMRI scanning. The MMSE and the WMS-R Logical Memory were utilized as a screening measure for cognitive impairments. The MMSE is a 30-question assessment of global cognitive status (Folstein et al., 1975), with a cut-off score of 24 out of 30. The WMS-R Logical Memory is a standardized assessment of narrative episodic memory (Wechsler, 1987). A short story is orally presented, and the examinee is required to recall the story immediately (Logical Memory I) and again 30 min later (Logical Memory II). Additionally, the Trail Making Test (TMT) A and B was used to evaluate executive function (Reitan, 1986). TMT contains two parts: part A is a numbered connect-the-dots task, and part B is a more complex connect-the-dots task that includes alternating letters and numbers.

### Materials

**FIGURE 1:**
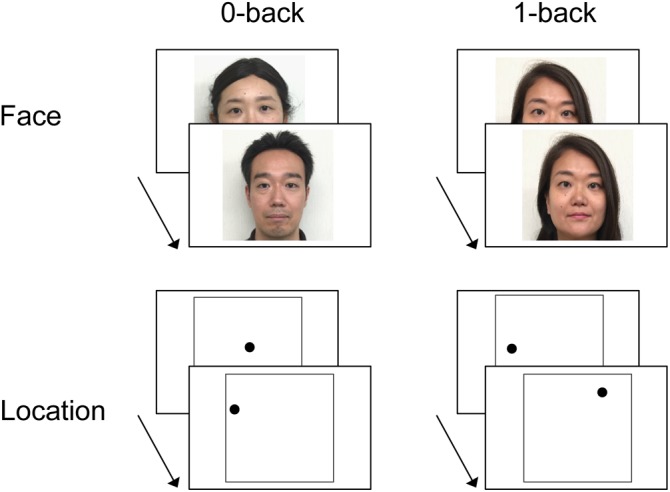
Example of stimuli and experimental design of the working memory (WM) tasks (*n*-back). In the face 0-back task, participants were asked to judge whether or not the face stimulus was female. In the location 0-back task, participants were asked to judge whether or not the dot was located in the center of the screen. In the 1-back task, participants were asked to judge whether or not the test item was identical to the one immediately preceding it (i.e., the face or the location of the dot presented in the last trial). Written consent was obtained from the individuals for the publication of their face images.

Examples of stimuli and the procedure are depicted in Figure 1. Two types of stimuli were used: faces and locations. Face stimuli were 52 colored photographs of faces with neutral expressions seen from a frontal viewpoint (26 females and 26 males). Location stimuli consisted of a black dot presented in one of various locations on the screen. Each face and each dot location was only presented once, except for the immediate repetitions to be detected in the face and location 1-back WM tasks, and the centered dot in the location 0-back WM task (see below for details).

Stimuli were projected onto a screen viewed by the subject via a mirror mounted on the scanner head coil. Each item (the face or the dot) appeared for 2000 ms, with a stimulus onset asynchrony (SOA) of 4000 ms. A black central fixation cross (+) was presented throughout the inter-item interval for 2000 ms. The WM tasks for face and location were conducted during separate fMRI runs. Each of the two runs contained eight task blocks (four blocks each for the 0-back and 1-back tasks) and four rest blocks. Each block lasted 32 s, and each task block consisted of eight trials (three possible correct “yes” responses per block; see below). The order of the blocks within a run was counterbalanced across participants.

### Procedure

Instructions and practice for the *n*-back tasks (0-back and 1-back task) were given prior to the scanning, outside the scanner. In the face 0-back task, participants were asked to judge whether or not the face stimulus was female. In the location 0-back task, participants were asked to judge whether or not the dot was located in the center of the screen. In the 1-back task, participants were asked to judge whether or not the test item was identical to the one immediately preceding it (i.e., the face or the location of the dot presented in the last trial). There was thus no WM required for the 0-back task, while the 1-back task required maintenance of information in WM for a short period of time. During rest, participants were instructed to relax and keep their attention on the central fixation. All responses were made with the index (“yes”) and middle (“no”) finger of the right hand using an MRI-compatible keypad. Participants were instructed to respond as quickly as possible without sacrificing accuracy.

### fMRI Scanning

Scanning was performed in a 3T Siemens Verio MR scanner (Siemens, Erlangen, Germany). Functional images were acquired with a T2^∗^-weighted axial echo-planer image (EPI) (TR, 2000 ms; TE, 25 ms; flip angle, 75°; FOV, 224 × 224; matrix size, 64 × 64). Each EPI volume was acquired in interleaved order and consisted of 39 axial slices (3.5 thick; in-plane resolution, 3.5 mm × 3.5 mm). fMRI data were acquired in two runs (197 volumes per run). The first five volumes of each run were discarded to allow for T1 equilibration. After the two functional runs, a whole-brain anatomical image was acquired using an axial T1-weighted, 3D magnetization-prepared rapid gradient echo (MP-RAGE) pulse sequence (FOV, 256 × 256; matrix size, 256 × 256; voxel size, 1 mm × 1 mm × 1 mm; 208 slices; axial acquisition).

### fMRI Data Analysis

Functional data preprocessing and statistical analyses were performed with Statistical Parametric Mapping (SPM12, Wellcome Department of Cognitive Neurology, London^2^) implemented in MATLAB R2012a (The Mathworks Inc., United States). Functional data were spatially realigned to the first volume of the series and then to the across-run mean volume, after which they were coregistered with the anatomical data. The anatomical data were normalized to MNI space using a unified segmentation procedure (Ashburner and Friston, 2005). The resulting deformation parameters were also applied to the functional data. The functional data were then resampled into 3 mm × 3 mm × 3 mm voxels and smoothed with an 8-mm full-width half-maximum (FWHM) Gaussian kernel.

Statistical analyses were performed in two stages of a mixed effects model. In the first stage, neural activity was modeled by a box-car function representing activity sustained throughout task blocks. These functions were then convolved with a canonical hemodynamic response function (HRF) to yield regressors in a general linear model that modeled the BOLD response for each task. We conducted two first-level analyses. In one analysis, four conditions were modeled: face 0-back, face 1-back, location 0-back, and location 1-back tasks. In the other analysis, two conditions were modeled: 0-back and 1-back tasks, each combining face and location WM tasks to maximize statistical power. Six regressors modeling movement-related variance (three rigid-body translations and three rotations determined from the realignment stage) and session-specific constant terms modeling the mean over scans were also used in the design matrix. Parameter estimates for events of interest were estimated using a general linear model. Non-sphericity of the error covariance was accommodated by an AR(1) model in which the temporal autocorrelation was estimated by pooling over suprathreshold voxels (Friston et al., 2002). Effects of interest were tested using linear contrasts of the parameter estimates. These contrasts were carried forward to a second stage of analysis treating subjects as a random effect. Two analyses of variance (ANOVA) were modeled: (1) a 2 × 2 × 2 mixed-design ANOVA with the factors age group (young–old and old–old adults), stimulus type (face and location), and task (0-back and 1-back tasks), and (2) a 2 × 2 mixed-design ANOVA with the factors age group (young–old and old–old adults) and task (0-back and 1-back tasks). Pair-wise contrasts derived from the ANOVA model were thresholded at *P* < 0.001, uncorrected, with an 18-voxel extent threshold. This cluster extent threshold was determined by a Monte Carlo simulation implemented in AlphaSim (Ward, 2000) to yield a corrected whole-brain cluster-wise significance level of *P* < 0.05. Regions demonstrating WM effects common to the two age groups were identified by a conjunction analysis. WM effects that differed according to age were identified by testing the interaction between age groups and WM effects. All coordinates are reported in MNI space.

In addition, using the MarsBaR toolbox (Brett et al., 2002), subject-specific parameter estimates for events of interest were extracted from a cluster that exceeded the statistical threshold mentioned above. The parameter estimates were averaged across voxels to yield a mean value for each cluster.

## Results

### Neuropsychological Performance

Demographic and neuropsychological data for the two age groups are summarized in Table 1. As is evident from the table, the old–old adults demonstrated lower performance on the TMT A and B.

**Table 1:** Demographic and neuropsychological data (mean, *SD*) for the two age groups.

	Young–old (*n* = 17)	Old–old (*n* = 16)
Age^∗∗^	66.1 (3.3)	79.4 (2.1)
Years of education	13.7 (1.9)	12.5 (2.4)
Mini Mental State Examination	28.0 (1.8)	27.4 (2.0)
Trail Making Test A^∗∗^	55.1 (12.2)	82.5 (20.3)
Trail Making Test B^∗∗^	92.8 (36.0)	131.8 (27.8)
WMS-R^1^ Logical Memory I composite score^2^	21.0 (6.1)	17.8 (4.6)
WMS-R^1^ Logical Memory II composite score^2^	16.6 (6.3)	13.5 (4.5)


*^*1*^Wechsler Memory Scale Revised. ^∗∗^P < 0.01. ^*2*^Group means (SD) provided in Kawano (2012), corresponding to the mean age of young–old and old–old groups, were as follows: young–old, Logical Memory I = 21.3 (7.1); II = 16.3 (6.8) and old–old, I = 17.0 (6.0); II = 11.9 (6.6).*

### Behavioral Performance

Table 2 shows the mean proportion of correct responses (accuracy) and the mean reaction times (RTs) of the young–old and old–old groups. A 2 × 2 × 2 ANOVA with the factors Group (young–old/old–old), Stimulus type (face/location) and Task (0-back/1-back) on proportion data revealed significant main effects of Group [*F*(1,31) = 19.85, *P* < 0.001], Stimulus type [*F*(1,31) = 11.48, *P* < 0.005] and Task [*F*(1,31) = 23.26, *P* < 0.001], along with significant interactions between Group and Task [*F*(1,31) = 8.54, *P* < 0.01] and between Stimulus type and Task [*F*(1,31) = 9.89, *P* < 0.005]. The former interaction indicates that accuracy differed between the two groups in the 1-back task (*P* < 0.001) but not in the 0-back task, and that 0-back and 1-back accuracy differed in the old–old group (*P* < 0.001) but not in the young–old group. The latter interaction reflects that the face condition was more difficult than the location condition in the 1-back task (*P* < 0.001), but not in the 0-back task.

**Table 2:** Mean (*SD*) proportion of correct responses (accuracy) and reaction times (RTs) of working memory tasks for the two age groups.

	Accuracy		RTs (ms)	
		
	Young–old	Old–old	Young–old	Old–old
**Face**				
0-back	0.99 (0.02)	0.96 (0.03)	1069.6 (92.9)	1113.0 (127.1)
1-back	0.94 (0.06)	0.86 (0.09)	1188.2 (163.6)	1344.4 (232.2)
**Location**				
0-back	0.98 (0.02)	0.97 (0.04)	1124.4 (136.6)	1110.1 (189.9)
1-back	0.99 (0.02)	0.91 (0.09)	1144.4 (148.7)	1273.1 (248.8)
**Collapsing across face and location**				
0-back	0.98 (0.01)	0.97 (0.03)	1097.0 (104.4)	1111.6 (143.9)
1-back	0.96 (0.03)	0.88 (0.08)	1166.3 (134.0)	1308.8 (227.5)


A 2 × 2 × 2 ANOVA on RTs gave rise to a significant main effect of Task [*F*(1,31) = 40.19, *P* < 0.001], and interactions between Group and Task [*F*(1,31) = 9.27, *P* < 0.005] and between Stimulus type and Task [*F*(1,31) = 11.25, *P* < 0.005]. The former interaction indicates that RTs differed between the two groups in the 1-back task (*P* < 0.05) but not in the 0-back task. The latter interaction reflects that responses in the face condition were slower than in the location condition in the 1-back task (*P* < 0.05), but not in the 0-back task.

### fMRI Findings

#### Stimulus Effects for Face and Location

**FIGURE 2:**
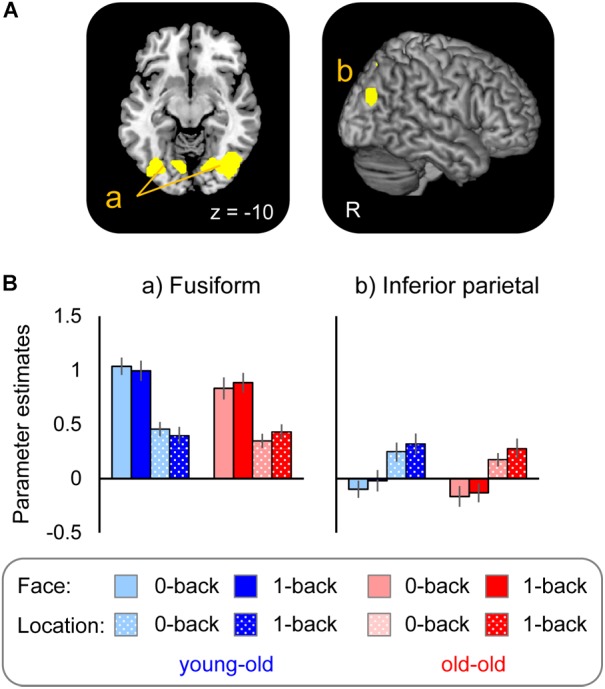
**(A)** Regions demonstrating selective stimulus effects for face and location common to the young–old and old–old groups. **(B)** Parameter estimates of the cluster for face and location WM tasks (0-back and 1-back tasks) in each group.

Regions selectively responsible for processing face (face > location) and location (location > face) stimuli common to the two age groups were identified in the right fusiform gyrus and the left lingual gyrus extending into the fusiform gyrus for face effects, and in the right lateral parietal cortex and the precuneus for location effects (see Figure 2A and Table 3). Mean parameter estimates, separated according to age group, are shown in Figure 2B. There were no regions where either stimulus effect differed between the groups.

**Table 3:** Brain regions showing stimulus effects for face and location common to the two age groups.

				Coordinates			
					
Brain region	L/R	BA	Number of voxels	*x*	*y*	*z*	*Z*-score
**Young–old = Old–old**			
*Face effects*			
Lingual gyrus	L	18	329	-9	-73	-4	4.50
Fusiform gyrus	R	37	666	42	-73	-10	5.75
*Location effects*			
Inferior parietal cortex	R	39	27	42	-76	26	4.38
Precuneus	R	7	31	18	-70	50	3.87


**Z-values refer to the peak of the activated cluster. L, left; R, right; BA, Brodmann’s area*.*

#### Working Memory Effects for Face and Location

##### Age-invariant WM effects

As is summarized in Table 4, WM effects for face common to the two age groups were identified in several regions, including the bilateral DLPFC (middle frontal gyrus), the inferior parietal cortex, the insula, the left ventrolateral prefrontal cortex (VLPFC; inferior frontal gyrus), the right medial frontal cortex, and the middle temporal gyrus. We failed to identify WM effects for location common to the two groups. This null result may be in part due to the fact that the proportion of correct responses was higher in location 1-back than in face 1-back tasks. However, this result does not necessarily indicate that the location 1-back task is not cognitively demanding. At a more liberal threshold (*P* < 0.005, uncorrected), there was an age-invariant WM effect for location in the bilateral insula (left: *x* = -30, *y* = 26, *z* = 2; right: *x* = 30, *y* = 26, *z* = 2) and in the right inferior parietal cortex (*x* = 51, *y* = -46, *z* = 50), overlapping regions identified in age-invariant face WM effects.

**Table 4:** Brain regions showing working memory effects for face and location common to the two age groups.

				Coordinates			
					
Brain region	L/R	BA	Number of voxels	*x*	*y*	*z*	*Z*-score
**Young–old = Old–old**							
*Face WM effects*			
DLPFC	L	44	52	-45	26	32	4.07
DLPFC	R	46	135	36	23	35	4.14
VLPFC	L	47	44	-36	47	-4	3.77
Medial frontal cortex	R	20	20	9	26	41	3.92
Insula	L	13	58	-30	26	2	5.15
Insula	R	13	67	30	29	-1	4.54
Middle temporal gyrus	R	21	20	66	-28	-10	3.98
Inferior parietal cortex	L	40	31	-39	-55	47	3.35
Inferior parietal cortex	R	39/40	186	42	-61	41	4.11
*Location WM effects*			
No suprathreshold clusters							


**Z-values refer to the peak of the activated cluster. L, left; R, right; BA, Brodmann’s area; DLPFC, dorsolateral prefrontal cortex; VLPFC, ventrolateral prefrontal cortex*.*

##### Age-related differences in WM effects

Brain regions where WM effects differed according to age, separately for face and location stimuli, are summarized in Table 5. Greater WM effects in the old–old than in the young–old group were identified in the right caudal DLPFC (superior frontal gyrus), both for face and location conditions. There were no regions where face or location WM effects were greater in the young–old than in the old–old group.

**Table 5:** Age-related differences (young–old vs. old–old) in working memory effects for face and location.

				Coordinates			
					
Brain region	L/R	BA	Number of voxels	*x*	*y*	*z*	*Z*-score
**Young–old > Old–old**							
*Face WM effects*			
No suprathreshold clusters							
*Location WM effects*			
No suprathreshold clusters							
**Old–old > Young–old**			
*Face WM effects*			
Caudal DLPFC	R	8/9	21	27	23	56	3.66
*Location WM effects*			
Caudal DLPFC	R	8/9	28	27	23	56	4.00
Inferior parietal cortex	L	40	44	-33	-58	41	3.51


**Z-values refer to the peak of the activated cluster. L, left; R, right; BA, Brodmann’s area; DLPFC, dorsolateral prefrontal cortex*.*

#### Working Memory Effects Collapsed Across Stimulus Types

Our primary interest was to investigate the age-related differences in WM effects associated with WM maintenance in general, independent of stimulus type. Since the age-related enhancement in WM effects was found in almost identical regions for face and location conditions, we collapsed across the two conditions within the respective task, to maximize statistical power.

##### Age-invariant WM effects

**FIGURE 3:**
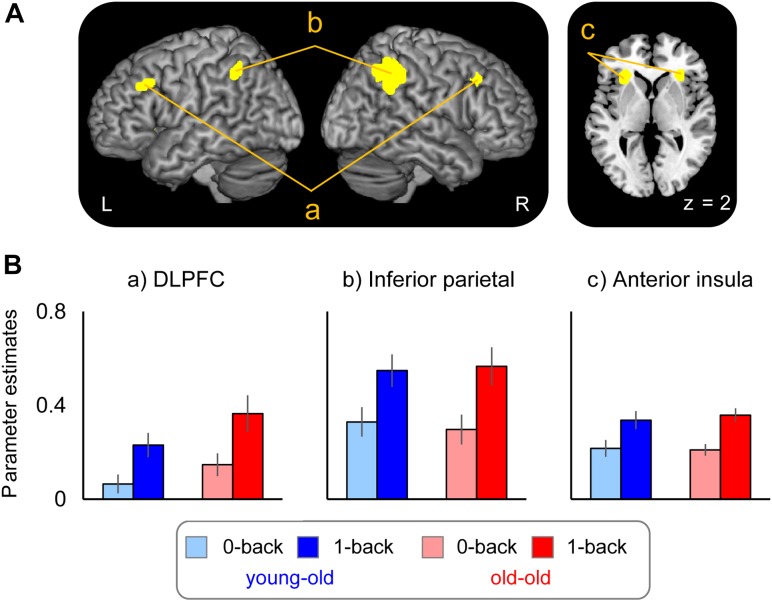
**(A)** Regions demonstrating working memory effects common to both the young–old and old–old group. **(B)** Parameter estimates of the clusters for the 0-back and 1-back task in each group.

WM effects common to the two age groups were identified in several regions, including the bilateral DLPFC (middle frontal gyrus), the inferior parietal cortex, and the insula. These results are summarized in Figure 3A and Table 6. Mean parameter estimates for each region, separated according to age group, are shown in Figure 3B.

**Table 6:** Brain regions showing working memory effects common to the two age groups.

				Coordinates			
					
Brain region	L/R	BA	Number of voxels	*x*	*y*	*z*	*Z*-score
**Young–old = Old–old**			
DLPFC	L	44	26	-48	26	35	3.95
DLPFC	R	46	26	36	23	38	3.64
Insula	L	13	47	-30	26	2	4.91
Insula	R	13	40	30	29	2	4.29
Inferior parietal cortex	L	40	18	-48	-49	47	3.52
Inferior parietal cortex	R	40	195	54	-46	44	4.18


**Z-values refer to the peak of the activated cluster. L, left; R, right; BA, Brodmann’s area; DLPFC, dorsolateral prefrontal cortex*.*

##### Age-related differences in WM effects

**FIGURE 4:**
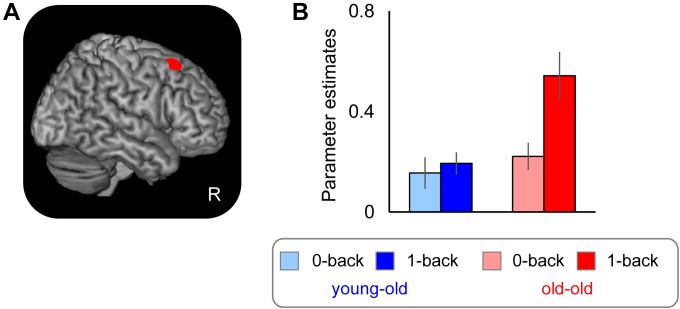
**(A)** Right caudal DLPFC, where WM effects were greater for the old–old than for the young–old group. **(B)** Parameter estimates of the clusters for the 0-back and 1-back task in each group.

Brain regions where WM effects differed according to age group are summarized in Figure 4A and Table 7. Greater WM effects in the old–old than in the young–old group were identified in a single cluster, the right caudal DLPFC (superior frontal gyrus). Mean parameter estimates, separated according to age group, are shown in Figure 4B. There were no regions where WM effects were greater in the young–old group than in the old–old group.

**Table 7:** Age-related differences (young–old vs. old–old) in working memory effects.

				Coordinates			
					
Brain region	L/R	BA	Number of voxels	*x*	*y*	*z*	Z-score
**Young–old > Old–old**							
No suprathreshold clusters							
**Old–old > Young–old**							
Caudal DLPFC	R	8/9	29	27	23	56	3.79


**Z-values refer to the peak of the activated cluster. L, left; R, right; BA, Brodmann’s area; DLPFC, dorsolateral prefrontal cortex*.*

Performance accuracy and RTs for the 1-back task significantly differed between the two groups. These results raise the question whether group differences in right DLPFC activity might simply be related to age differences in task performance. We therefore repeated the interaction analyses using ANOVA with accuracy and RTs as covariates to test whether the group differences in WM effects would persist. Again, we identified the right caudal DLPFC where WM effects were greater in the old–old group (*x*, *y*, *z* = 27, 23, 56; *Z* = 3.67; 18 voxels).

#### Relationship Between Over-Recruitment in Working Memory Effects and Performance

**FIGURE 5:**
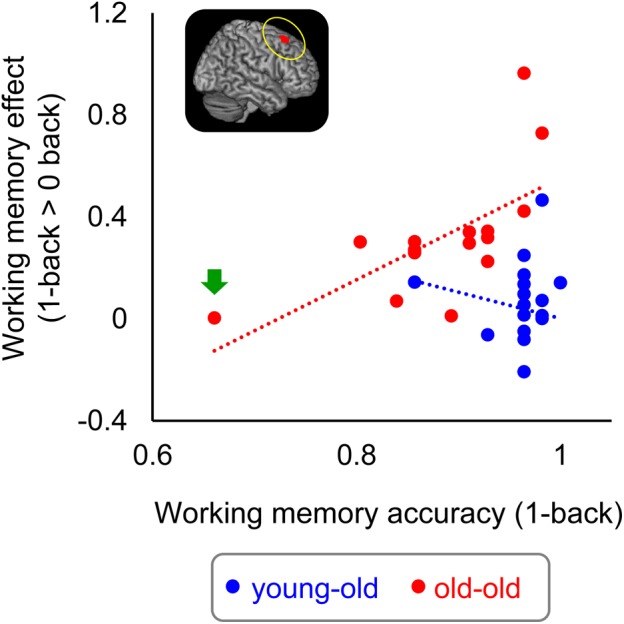
Scatterplots illustrating the relationship between WM effects in the right caudal DLPFC and proportion of correct responses in the 1-back task in each group. One outlying old–old adult is indicated by a green arrow (see text).

As is noted in the Section “Introduction,” it has been debated whether the age-related over-recruitment in WM effects serves as a compensatory mechanism for age-related decline in WM performance (Cabeza, 2002; Davis et al., 2008; see also Reuter-Lorenz and Cappell, 2008). We therefore computed across-participants correlations between the magnitudes of over-recruitment in the right caudal DLPFC identified in the foregoing analysis (see section “Age-Related Differences in WM Effects” and Table 7) and WM performance (accuracy in the 1-back task), separately for each group. Significant positive correlations were evident in the old–old group (*r* = 0.65, *P* < 0.01) but not in the young–old group (*r* = -0.16, *P* = 0.54). There were no outlying participants who showed WM effects 2 SDs above/below the group mean. We found one old–old adult who performed 2 SDs below the group mean in the 1-back task; however, the significant correlation was still evident when this participant was omitted (*r* = 0.63, *P* < 0.05). Figure 5 illustrates the relationship between the participants’ WM effects (1-back > 0-back) and accuracy in the 1-back task for the two groups.

#### Age-Related Differences in Working Memory Effects Between the Young–Old and High-Performing Old–Old Groups

In light of the argument that the interpretation of age-related differences in brain activity is often confounded with age-related differences in task performance (see Rugg and Morcom, 2005 for details), we performed an additional analysis comparing two age groups with the same level of WM performance. We separated the participants in the old–old group into two subgroups (high and low performers) based on a median split of their accuracy in the 1-back task, and contrasted the WM effects of the young–old (*n* = 17) and a “high-performing” old–old group (*n* = 8) (resulting mean of 1-back accuracy/RTs: young–old = 0.96/1166.3 ms; high-performing old–old = 0.94/1195.4 ms; no significant group differences, *P* > 0.1). The aim of this analysis was to investigate whether the age-related right caudal DLPFC over-recruitment identified above (see section “Age-Related Differences in WM Effects”) can also be detected when WM performance is matched between the two age groups.

**FIGURE 6:**
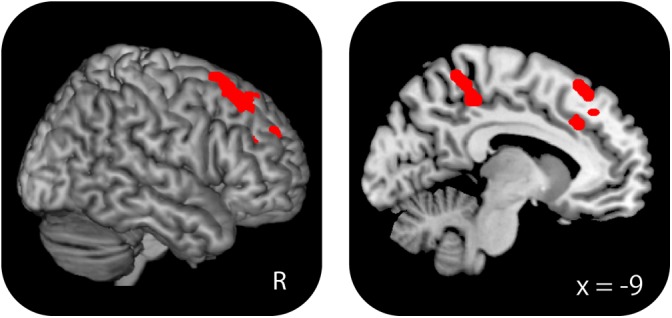
Regions where WM effects were greater for the high-performing old–old than for the young–old group. These two groups were equivalent in terms of WM performance.

Greater WM effects in the high-performing old–old than in the young–old group were identified in the right caudal DLPFC (superior frontal gyrus), the DLPFC (middle frontal gyrus), the left medial frontal cortex, and the precuneus (Table 8 and Figure 6). We identified an overlapping region in the right caudal DLPFC between the current (high-performing old–old vs. young–old) and the foregoing results (old–old vs. young–old; section “Age-Related Differences in WM Effects”) (*x*, *y*, *z* = 33, 26, 56; *Z* = 4.62; 29 voxels; *P* < 0.05, small-volume corrected). Again, there were no brain regions where WM effects were greater for the young–old than for the high-performing old–old group.

**Table 8:** Age-related differences (young–old vs. high-performing old–old) in working memory effects.

				Coordinates			
					
Brain region	L/R	BA	Number of voxels	*x*	*y*	*z*	*Z*-score
**Young–old > High-performing old–old**							
	No suprathreshold clusters							
**High-performing old–old > Young–old**							
Caudal DLPFC	R	8/9	219	33	26	56	4.53
DLPFC	R	46	63	24	47	29	3.80
Medial frontal cortex	L	9/32	52	-3	32	35	3.90
Medial frontal cortex	L	8	23	-9	32	53	3.71
Precuneus	L	7	41	-12	-46	62	3.45


**Z-values refer to the peak of the activated cluster. L, left; R, right; BA, Brodmann’s area; DLPFC, dorsolateral prefrontal cortex*.*

## Discussion

The primary interest was to evaluate whether an age-related increase in WM effects is observed in advanced old age. Consistent with prior studies that reported increased WM effects when contrasting older and younger adults (Rajah and D’Esposito, 2005; Reuter-Lorenz and Sylvester, 2005; Spreng et al., 2010), we identified age-related over-recruitment in the right caudal DLPFC. Our secondary interest was to investigate whether this over-recruitment reflects the engagement of compensatory responses to maintain performance. We found that over-recruitment effects in the right caudal DLPFC were positively correlated with performance in old–old adults but not in young–old adults. In the following, we discuss the implications of these findings and their possible links to previous studies of age-related differences in WM.

### Behavioral Findings

There were significant group differences in proportions of correct responses and RTs for the 1-back task, but performance measures for the 0-backs task did not significantly differ. These results are consistent with previous studies suggesting that WM maintenance abilities are markedly declined in individuals around the age of 80 (Park et al., 2002). A more important finding is that old–old adults showed a higher variability in WM performance. The accuracy ranges for the 1-back task was 0.66–0.98 for the old–old group and 0.86–1.0 for the young–old group. In general, individual differences in cognitive decline become more pronounced with advanced age (de Frias et al., 2007; Habib et al., 2007; see also Morrison and Baxter, 2012; Nyberg et al., 2012). This variability allowed us to examine direct relationships between WM cortical activity and performance, and also to perform separate analyses that compare young–old and high-performing old–old adults (see section “Results”) with equivalent WM performance. Thus, although WM performance significantly differed between the young–old and the old–old group, we were able to examine whether group differences in WM effects reflect age-related differences in the recruitment of neural networks during WM tasks.

### fMRI Findings

#### Stimulus Effects for Face and Location Common to the Young–Old and Old–Old Groups

Age-invariant stimulus effects for face were identified in the bilateral fusiform gyrus, while those for location were identified in the right inferior parietal cortex and the precuneus. Numerous previous studies have reported selective responses associated with visual processing of face information in the fusiform gyrus (Kanwisher et al., 1997; Haxby et al., 2000) and of location information (especially for egocentric spatial information) in the lateral parietal cortex (Andersen et al., 1985; Stark et al., 1996). More importantly, no clusters were identified where selective responses for face or location differed between the two groups. These results were inconsistent with prior findings from studies comparing younger and older adults that identified age-related reduction in activity in posterior regions (such as visual areas) during visual processing (Ross et al., 1997; Dennis and Cabeza, 2008). Thus, the present findings indicate that neural activity associated with visual processing of face and location representations is not affected by advanced aging. Supporting evidence comes from the finding that behavioral measures (accuracy and RTs) for face and location 0-back tasks are equivalent between the two groups.

#### Working Memory Effects Common to the Young–Old and Old–Old Groups

Age-invariant WM effects were identified in the bilateral DLPFC (middle frontal gyrus) and the inferior parietal cortex. These regions are widely known to be critical for WM (e.g., Owen et al., 2005; Rottschy et al., 2012) and form part of the “core” WM network (Rottschy et al., 2012). One of the most intriguing findings is that both the young–old and the old–old group showed WM effects in these regions bilaterally. When comparing younger and older adults, older adults tend to recruit PFC regions contralateral to those most active in younger adults, yielding a more bilateral pattern of PFC activation (cf. the HAROLD; Cabeza, 2002). For example, Reuter-Lorenz et al. (2000) used a task in which participants maintained four letters or dot locations in WM and then compared those to a probe item. Younger adults showed activity in the left PFC during a verbal WM task and in the right PFC during a non-verbal WM task, whereas older adults showed bilateral PFC recruitment during both WM tasks. In the same line of study, Reuter-Lorenz et al. (2001) identified a similar pattern of bilateralization of parietal activity in older adults, and left lateralization in younger adults during a verbal WM maintenance task. Considering this evidence, bilateral recruitment of the DLPFC and the parietal regions in our two older groups is an expected finding, resulting from the bilateralization tendency of brain activation in older adults.

Age-invariant WM effects were also evident in the bilateral anterior insula. A meta-analysis found that this region is often reported in WM studies (e.g., Owen et al., 2005; Rottschy et al., 2012). The anterior part of the insula is a component of the “salience network,” which is thought to be particularly involved in detecting novel salient events and in initiating control signals to engage brain regions mediating attention, WM, and higher order cognitive processes, such as the fronto-parietal network (Seeley et al., 2007; Menon and Uddin, 2010). WM effects in this region might thus reflect the allocation of processing resources to the detection of salient aspects of events (e.g., novel features of an item), and, once a salient event is detected, to the facilitation of access to core WM resources to guide appropriate responses.

#### Age-Related Over-Recruitment in Working Memory Effects

Compared to the young–old group, the old–old group showed increased WM effects in the right caudal DLPFC (superior frontal gyrus). This result is in part consistent with prior studies that reported age-related prefrontal over-recruitment when comparing older and younger adults. As mentioned above, however, older adults tend to additionally recruit PFC regions contralateral to those most active in younger adults (Cabeza, 2002). Based on this evidence, we would have expected to detect left instead of right prefrontal over-recruitment in old–old compared to young–old adults, since we employed a visual WM task. In contrast, however, we found age-related over-recruitment in the right PFC. In addition, the caudal DLPFC over-recruitment found here did not couple with an age-related reduction in the recruitment of posterior regions involved in perceptual processes (cf. the PASA; Davis et al., 2008). There were no clusters where selective responses for face or location differed between the two groups. These results suggest that the tendency to shift the load of cognitive processing from unilateral to bilateral PFC, and from posterior to PFC in older adults might reach a plateau around the age of mid-sixties (i.e., in our young–old group and in older adults in other WM studies).

Over-recruitment in the caudal DLPFC reflects that old–old adults rely on additional cognitive processes mediated by this region to a greater extent than do young–old adults for WM maintenance. Converging evidence suggests that the caudal DLPFC is implicated in updating the focus of attention toward representations in WM (Roth et al., 2006; Bledowski et al., 2009; see Wager and Smith, 2003; Bledowski et al., 2010 for reviews). As is briefly explained in the Introduction, the focus of attention contributes to holding task-relevant information in WM that can be used in ongoing cognitive tasks (Cowan, 1995; Oberauer, 2002; Fuster, 2009; D’Esposito and Postle, 2015; Eriksson et al., 2015). In a 1-back task, items are continuously presented, and each presentation evokes related internal representations; the items are actively maintained via focusing sustained attention and updated when the information is no longer needed to achieve the task requirements. Old–old adults may assign more neurocognitive resources to the shift in attention that enables them to effectively update representations in WM from old, no longer task-relevant information to newer, more relevant information, which may reflect the increased caudal DLPFC activation in old–old adults we observed here.

In terms of the effect of advanced aging, Wang et al. (2009) focused on episodic memory and compared retrieval-related activity between young–old and old–old groups using a yes/no recognition memory task. In this study, participants studied a series of pictures, and later indicated whether each picture was old or new during the scanning. Recognition performance was matched between groups by modifying the number of presentations in the study phase (once or twice). The authors found age-invariant old > new effects in the parietal and prefrontal cortex where such effects have been reported in prior studies in younger and older adults (e.g., Daselaar et al., 2003; Morcom et al., 2007; Duarte et al., 2010). No clusters were identified with greater effects in the old–old group, while the medial parietal region showed greater effects in the young–old group.

Currently, there is no plausible explanation for why Wang et al.’s (2009) and our results differ in terms of age-related over-recruitment in brain activation in advanced aging. Performance accuracy in old–old adults is high in both studies [Wang et al.’s (2009) recognition task, old items: 88.9%; our 1-back task: 88.4%], indicating that both tasks imposed a relatively low level of cognitive demand on participants. If tasks with low cognitive demand always induced greater brain activation in older than in younger adults with matched task performance (e.g., Cabeza et al., 2004; Mattay et al., 2006; see also Grady, 2008; Grady, 2012), a dissociation of findings between the two studies would not be observed. One possible explanation for this dissociation is the difference in cognitive processes required for each task. Compared to the recognition task, as discussed previously, the 1-back task requires continuous attention toward the items and their representations until the task is finished. The processes involved in this kind of continuous attention are highly mediated by the DLPFC, which is the region where age-related over-recruitment is most prominently observed (c.f. Rajah and D’Esposito, 2005; Reuter-Lorenz and Sylvester, 2005; Spreng et al., 2010). Combining findings from both studies indicates that, at least in the case of advanced aging, whether age-related cortical over-recruitment occurs depends on the cognitive processes that are necessary for task execution.

Another possible explanation is the use of different experimental fMRI designs [Wang et al.’s (2009) study: event-related vs. our study: blocked design]. Consistent with this possibility, most prior studies of episodic and WM in young and older adults that identified age-related over-recruitment in brain activity employed a blocked design (e.g., Rypma et al., 2001; Nyberg et al., 2014). It is noteworthy that one mixed event-related block-design fMRI study using a 1-back WM task found differences in age-related over-recruitment between sustained activity during task blocks and transient activity related to target items (Grady et al., 2008). In this study, enhanced PFC activation for older compared to younger adults was identified during sustained but not during transient activity. However, as age-related over recruitment of each memory effect has also been identified in event-related fMRI studies (e.g., Daselaar et al., 2003; Cabeza et al., 2004; Morcom et al., 2007; Cappell et al., 2010; Duarte et al., 2010), the effect of experimental design on neural correlates of advanced aging needs to be further examined.

An alternative explanation for the differing results is that Wang et al.’s (2009) study employed much older old–old adults (84–96 years old) than we did in the current study (77–82 years old). As Wang et al. (2009) also pointed out, older persons aged ≥85 years might reach the limit of their ability to recruit additional neural resources as compensatory responses against a continuing cognitive decline. Since there is currently not enough evidence to support or dismiss this hypothesis, future studies will be required.

#### Age-Related Over-Recruitment in WM Effects and Compensation

Age-related over-recruitment in WM effects in the caudal DLPFC showed a positive correlation with WM performance, but only in the old–old group. In addition, the group differences in WM effects in this region remained after covariate adjustment of performance accuracy and RTs in the 1-back task. Moreover, this region showed greater activity when we compared WM effects between two age groups with matched performance (i.e., high-performing old–old > young–old group). Thus, although the argument has been put forward that the interpretation of age-related differences in brain activity is often confounded with age-related differences in task performance (Rugg and Morcom, 2005), the present results clearly confirm that over-recruitment in the caudal DLPFC reflects age-related changes in WM effects and serves as a compensatory mechanism for maintaining WM performance in old–old adults. As discussed in the Introduction, individual differences in cognitive decline become more pronounced with advanced age. The present results further indicate that such a variation may occur as a result of individual differences in the ability to utilize additional PFC resources (e.g., the caudal DLPFC in the present study) that permit some older adults to maintain higher performance than others, to compensate for a continuing decline in neural efficiency. We note, however, that our results are correlational, preventing us from drawing firm conclusions concerning brain-behavior causality. An intervention study of WM training would be necessary to provide better understanding of a causal relationship between WM performance and reorganization of PFC activity in advanced aging (e.g., Brehmer et al., 2011; Nishiguchi et al., 2015; Adnan et al., 2017; Vermeij et al., 2017; Iordan et al., 2018).

Another piece of evidence supporting the idea that caudal DLPFC over-recruitment plays a role as part of a compensatory network comes from a meta-analysis of WM studies in healthy adolescents (10–17 years) and young adults (18–30 years) (Andre et al., 2016). Regions where WM-related activity increased with age were identified in a part of the core WM network (the DLPFC and the lateral parietal cortex), while regions where activity decreased with age were evident in the caudal DLPFC along with the post central and cingulate gyrus. The authors raised the possibility that regions showing activity reduction with age form part of a compensatory network that supports the still maturing core WM network. Thus, while the direction of the effects this study reports seems to be opposite to our findings, a dynamic relationship between aging/development of the core WM network and engagement of the caudal DLPFC in WM can be assumed. That is, in individuals around 80 years of age (i.e., our old–old group), higher engagement of the caudal DLPFC might be required, due to the neurocognitive decline of the core WM network, whereas in adolescence, involvement of the caudal DLPFC might be less essential as the core WM network matures.

### Study Limitations

The results of this study should be interpreted in the context of several limitations. The first limitation concerns the use of a blocked design, which did not allow us to further separate each trial into different component processes, such as encoding, maintenance, or retrieval from WM. Although we speculate that the over-recruitment in the caudal DLPFC might reflect the greater reliance of old–old adults on updating focused attention toward representations in WM, this hypothesis can only be confirmed by the use of an event-related design.

Secondly, as is mentioned in the Introduction, previous functional imaging studies have often reported enhanced PFC activity at low WM loads in older adults, while older adults tend to show reduced PFC activity compared to younger adults at high WM loads (e.g., 2-back task; Mattay et al., 2006; Nyberg et al., 2009; Heinzel et al., 2017). We did not include a 2-back task, because data from a previous behavioral study by our group (Kawagoe and Sekiyama, 2014) revealed that 2-back tasks for face and location may be too cognitively demanding for old–old adults. The use of a task with higher WM load would allow us to investigate the relationship between the over-recruitment of PFC activity and behavioral performance in a more direct way (cf. compensation-related utilization of neural circuits hypothesis, CRUNCH; Reuter-Lorenz and Cappell, 2008). Conclusions drawn from the present results should therefore be restricted to tasks with low WM load.

Thirdly, the sample size was relatively small (17 young–old and 16 old–old adults), and a greater number of participants in each group would lead to higher statistical power.

Fourthly, this study is limited by the shortcomings of cross-sectional experimental designs, namely cohort effects. The proportion of the elderly population with MCI or dementia markedly differs in groups of elderly people in their 60s and 80s in Japan (11.3 and 44.7%, respectively; Asada, 2013). We can thus not rule out the possibility that the age-related differences observed here reflect the consequences of contrasting two groups with different traits of resistance to cognitive impairment (see Rugg, 2016 for details). This possibility can only be ruled out by using a longitudinal experimental design.

Finally, there is a possibility that the age-related difference in DLPFC activity shown in the present study is confounded by age-related alterations in cerebrovascular dynamics (e.g., reduced vascular reactivity or pathology), which are known to affect the BOLD signal (cf. D’Esposito et al., 2003). However, a study that compared memory-encoding fMRI responses between younger and older adults found that age-related enhancement in frontal activity was still observed after removing the influence of changes in vascular reactivity (Liu et al., 2013).

## Conclusion

The present fMRI studysuggests that an age-related increase in PFC activity associated with WM effects occurs in advanced old age, similar to earlier findings on WM effects from studies comparing younger and older adults. Moreover, although the functional significance of the age-related over-recruitment still remains under debate, our findings provide strong evidence that this over-recruitment serves a compensatory function in mediating WM performance in adults of advanced age. Our results support the compensation account of cognitive aging (stating that the recruitment of additional PFC regions is beneficial to performance) and indicate that this theory can also be applied to adults of advanced age.

## Author Contributions

MS, TK, SN, NA, YO, RN, MY, SY, and KS study conception and design. MS, TK, SN, NA, YO, RN, and KA data acquisition. MS data analysis and interpretation, and drafting of the manuscript. TK, SN, NA, YO, RN, KA, MY, SY, and KS critical revision of the manuscript.

## Acknowledgments

We thank Dr. Mamoru Hashimoto forvisual inspection of abnormalities in anatomical brain MRI. This study was conducted using the MRI scanner and related facilities of the Kokoro Research Center, Kyoto University.

## Conflict of Interest Statement

The authors declare that the research was conducted in the absence of any commercial or financial relationships that could be construed as a potential conflict of interest.

## Footnotes

1 http://esa.un.org/unpd/wpp

2 http://www.fil.ion.ucl.ac.uk/spm
